# A Molecular Screening of Mosquitoes (Diptera: Culicidae) for Flaviviruses in a Focus of West Nile Virus Infection in Northern Iran

**Published:** 2019-12-31

**Authors:** Vahideh Moin-Vaziri, Remi N Charrel, Mehdi Badakhshan, Xavier de Lamballerie, Nourina Rahbarian, Mulood Mohammadi Bavani, Shahyad Azari-Hamidian

**Affiliations:** 1 Department of Parasitology and Mycology, School of Medicine, Shahid Beheshti University of Medical Sciences, Tehran, Iran; 2 Unité des Virus Emergents (UVE: Aix Marseille Univ, IRD 190, INSERM 1207, IHU Méditerranée Infection), Marseille, France; 3 Department of Medical Entomology and Vector Control, School of Public Health, Urmia University of Medical Sciences, Urmia, Iran; 4 Department of Health Education, Research Center of Health and Environment, School of Health, Guilan University of Medical Sciences, Rasht, Iran

**Keywords:** *Aedes*, *Anopheles*, *Coquilletidia*, *Culex*, *Flavivirus*

## Abstract

**Background:**

Mosquito-borne arboviruses such as West Nile (WN), dengue, Rift Valley fever, and Sindbis viruses are reported in Iran, but large-scale studies have not been performed on mosquitoes to find their vectors. A molecular study of the adult mosquitoes (Diptera: Culicidae) for flaviviruses was carried out in a focus of WN infection, Guilan Province, northern Iran.

**Methods:**

Mosquito collections were carried out in five stations of two counties (Anzali and Rasht) using light traps, hand catch by manual aspirators and night landing catch during August–September 2013 and 2014. Molecular screening of WN virus and more widely for *Flavivirus* RNA was carried out using a specific PCR technique.

**Results:**

In total, 1015 adult mosquitoes were collected including eight species representing four genera. The most prevalent species were *Aedes vexans* (33.2%), *Culex tritaeniorhynchus* (22%), *Cx. pipiens* (20.7%), and *Anopheles maculipennis* s.l. (15.6%). Molecular screening was carried out on the 1015 mosquitoes after they were organized as 38 pools according to sex, species and trapping location. None of the pools were positive.

**Conclusion:**

Surveillance should be continued while increasing the sampling campaigns due to the presence of wetlands in the region and abundant species which are considered as vectors, feeding on both birds and humans.

## Introduction

Based on the latest classification, mosquitoes (Diptera: Culicidae) include two subfamilies, 11 tribes, 41 or 113 genera (depending on the generic classification of the tribe Aedini) and 3563 species (1). Mosquitoes are involved in the transmission of several arboviruses belonging to different families such as Flaviviridae, Phenuiviridae and Togaviridae (2). The family Flaviviridae comprises 58 viruses that are of greatest concerns for human health. The genus *Flavivirus* includes 53 virus species, of which 39 are transmitted by mosquitoes or ticks (3). West Nile virus (WNV) (Flaviviridae: *Flavivirus*) is distributed in Eurasia, Africa, North and Central America and Australia. Mosquitoes are the principal vectors of the virus and some virus isolations have been reported from soft and hard ticks (Arachnida: Ixodida). Wild birds, especially wet-land species, are the principal vertebrate hosts and also the virus has been isolated from mammals and frogs (4).

The most recent checklist of Iranian mosquitoes includes seven genera and 64 species (5, 6). Subsequently, *Anopheles superpictus* was found to be a complex including two species in Iran based on the internal transcribed spacer 2 (ITS2) sequences of rDNA (7) which later were listed as species A and B (8). A new species of the *An. hyrcanus* group (*An. hyrcanus* spIR) was recognized from southwestern Iran, also based on ITS2 sequence data (9). More recently, the occurrence of *Aedes albopictus* [*Stegomyia albopicta*] and *Ae. unilineatus* [*Stegomyia unilineata*] in southeastern Iran was reported (10, 11). Finally, *Orthopodomyia pulcripalpis* was reported in northern Iran (12).

Several pathogens, which are known to be transmitted by mosquitoes, are reported in Iran such as *Dirofilaria immitis* and *D. repens* (13), different *Plasmodium* spp. (9, 14), West Nile, dengue viruses (DENV) (Flaviviridae: *Flavivirus*), Sindbis virus (SINV) (Togaviridae: *Alphavirus*) and Rift Valley Fever virus (RVFV) (Phenuiviridae: *Phlebovirus*) (15–20). It is noteworthy that there is the possibility of some other mosquito-borne arboviral outbreaks including Japanese encephalitis virus (JEV) (Flaviviridae: *Flavivirus*) in the World Health Organization Eastern Mediterranean Region (21). Recently, infection to WNV has been confirmed by the polymerase chain reaction (PCR) technique in *Ae. caspius* [*Ochlerotatus caspius*] and *Culex pipiens* in northwestern and northern Iran, respectively (22, 23).

West Nile virus is reported in 26 provinces (Out of 31) of Iran in horses (17, 24, 25), humans (15–17, 26–30) and birds (31). Guilan Province in Caspian Sea littoral, northern Iran, with vast wetlands is probably one of the foci of WNV where the infection is found in humans (1.4–10%) (16, 17, 26), horses (2.2–25%) (17, 24) and birds (especially common coot as a main reservoir) (62.7%) (31).

Surprisingly, despite the obvious importance in the emergence of viral diseases caused by flaviviruses such as WNV and DENV, little data is published about their mosquito vectors in Iran and large-scale studies have not been performed. Also, there is no official vector surveillance for WNV. The aim of the present investigation was to inventory mosquitoes in Guilan Province and to screen these mosquitoes for WNV and more widely for flaviviruses using both virus-specific real-time RT-PCR and a real-time pan-flavivirus RT-PCR, respectively.

## Materials and Methods

### Study areas

**Fig. 1.**
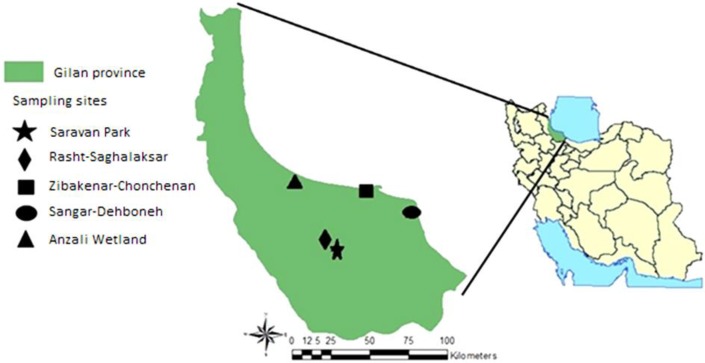
Map showing the sampling sites (Marked by signs) and the geographical location of Guilan Province in Iran

**Fig. 2.**
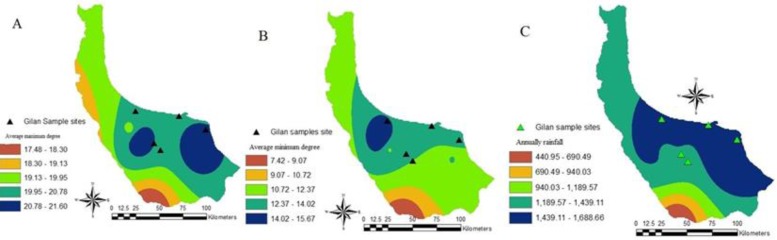
Sampling sites over the layers of different climatic condition of Guilan Province (A: Average maximum degree, B: Average minimum degree C: Annual rainfall)

The study took place in Guilan Province (36°33′–38°27′ N and 48°32′–50°36′ E) during August–September 2013 and 2014. The province is located along the Caspian Sea and surrounded by Mazandaran Province in the east, Ardebil in the west and Zanjan and Qazvin in the south. It also borders the Republic of Azerbaijan in the north, as well as Russia across the Caspian Sea. The province is humid with mean annual rainfall ranging 1000–2000mm. Collections were carried out in five sites including Saghalaksar of Rasht, Dehboneh of Sangar, Saravan Park and Chonchenan of Zibakenar (all in Rasht County) and Ghazian alongside Anzali Wetland, which is one of the few international Iranian wetlands (Anzali County) (Fig. 1). The collection sites are shown over the layers of minimum and maximum degree of temperature and annual rainfall of Guilan Province in Fig. 2.

### Mosquito sampling and processing

Adult mosquitoes were collected by using CDC miniature light traps, operating overnight from sunset to sunrise, i.e. from 18:00PM to 6:00AM, hand catch by manual aspirators from hen shelters, barns and bathrooms, and night landing catch from human bate. Specimens were transferred alive to the laboratory where identified using morphological-based keys (6) after anaesthetizing using an ice bag then transferred to the portable nitrogen tank.

### Pooling of mosquitoes for viral RNA testing

The mosquitoes were grouped into the pools according to sex, species and trapping location. Pools were homogenized in a final volume of 600μL as previously described (32). A 200μL volume was used for viral nucleic acid (NA) extraction using the BioRobot EZ1-XL Advanced system (Virus Extraction Mini Kit, Qiagen).

### Real-time RT-PCR for specific detection of West Nile virus RNA

FiveμL of NA was used for RT-PCR. Sense (ProC-F1: CCTGTGTGAGCTGACAAACTT AGT) and reverse (ProC-R: GCGTTTTAGC ATATTGACAGCC) primers were combined with the fluorogenic TaqMan probe (ProC-TM: 6FAM-CCTGGTTTCTTAGACATCGAGAT CTTCGTGC TAMRA), and used with the Go Taq Probe 1-Step RT-qPCR (Promega) as previously reported (33). This assay was developed and is routinely used by the French Reference Centre for Arboviruses.

### Molecular detection of *Flavivirus* RNA

Total nucleic acid extraction was conducted by using a Biorobot EZ1, with virus Mini Kit v2.O (Qiagen). Another 5μl aliquot of NA was used in a one-step Real-Time Quanti-Tec SYBER-GREEN RT-PCR assay (Qiagen) as previously described (34). This assay located in a highly conserved region of the polymerase gene allows detection of all recognized flaviviruses, species identification is then achieved by sequencing the PCR product and comparing the sequence using BLAST NCBI program software and database.

## Results

A total of 1015 adult mosquitoes were collected from Guilan Province including eight species representing four genera as follow: three species within subfamily Anophelinae (*An. maculipennis* s.l., *An. pseudopictus*, *An. sacharovi*) and five species within subfamily Culicinae (*Ae. vexans* [*Aedimorphus vexans*], *Coquillettidia richiardii*, *Cx. pipiens*, *Cx. theileri*, *Cx. tritaeniorhyncus*) (Table 1). The largest species diversity was observed in Ghazian, alongside Anzali Wetland, where all eight species were recorded. The most prevalent species were *Ae. vexans* (33.2%), *Cx. tritaeniorhynchus* (22 %), *Cx. pipiens* (20.7%), and *An. maculipennis* s.l. (15.6%), respectively. *Culex pipiens* was collected from all five sites followed by *Ae. vexans* and *Cx. tritaeniorhynchus* (four sites) and *An. maculipennis* s.l. (three sites) (Table 1). The composition percentages of species based on the collection methods were as follow: *An. maculipennis* s.l. [91.8% by night landing catch (NLC), 8.2% by aspirator (AS), *An. pseudopictus* (90.9% by NLC, 9.1% by AS), *An. sacharovi* (100% by AS), *Ae. vexans* (96.4% by NLC, 3.6% by AS), *Cq. richiardii* (100% by NLC), *Cx. pipiens* [55.7% by AS, 37.6% by NLC, 6.7% by light trap (LT)], *Cx. theileri* (100% by AS), *Cx. tritaeniorhynchus* (78.1% by AS, 21.9% by NLC). The 1015 mosquitoes were grouped into 38 pools according to sex, species and trapping location. None of the 38 pools tested by real-time RT-PCR either for flaviviruses or more specifically for West Nile virus was positive.

**Table 1.** Details of collected specimens based on study areas and collection methods

**Species**	**n**	**Collected sites**	**Collection methods**	**Year**	**total**
***An. maculipennis* s.l.**	11	Rasht-Saghalaksar	AS^a^	2013	158 (15.6%)
	1	Anzali Wetland	NLC^b^	2013	
	2	Sangar-Dehboneh	AS	2013	
	144	Anzali Wetland	NLC	2014	

***An. pseudopictus***	4	Anzali Wetland	AS and NLC	2013	22 (2.2%)
	4	Anzali Wetland	NLC	2014	
	14	Saravan Park	NLC	2014	

***An. sacharovi***	18	Anzali Wetland	AS	2014	18 (1.8%)

***Ae. vexans***	1	Rasht-Saghalaksar	AS	2013	330 (32.5%)
	8	Zibakenar-Chonchenan	NLC	2013	
	57	Anzali Wetland	AS and NLC	2013	
	255	Anzali Wetland	NLC	2014	
	9	Saravan Park	NLC	2014	

***Cq. richiardii***	11	Anzali Wetland	NLC	2013	35 (3.4%)
	24	Anzali Wetland	NLC	2014	

***Cx. pipiens***	84	Rasht-Saghalaksar	AS	2013	210 (20.7%)
	30	Zibakenar-Chonchenan	LT^c^ and AS	2013	
	9	Anzali Wetland	AS	2013	
	8	Sangar-Dehboneh	AS	2013	
	51	Saravan Park	NLC	2013	
	26	Anzali Wetland	NLC	2014	
	2	Saravan Park	NLC	2014	

***Cx. theileri***	18	Anzali Wetland	AS	2014	18 (1.8%)

***Cx. tritaeniorhyncus***	30	Rasht-Saghalaksar	AS	2013	224 (22.0%)
	143	Anzali Wetland	AS	2013	
	2	Sangar-Dehboneh	AS	2013	
	29	Anzali Wetland	NLC	2014	
	20	Saravan Park	NLC	2014	

**Total**			1015 (100%)		

a AS = Aspirator,

b NLC = Night Landing Catch,

c LT = Light Trap)

## Discussion

In total, eight different species of mosquitoes were collected during this study, all found in Guilan Province before (9, 35). The most prevalent and widespread species of the present study i.e. *Ae. vexans*, *Cx. tritaeniorhynchus*, *Cx. pipiens* and *An. maculipennis* s.l. were also found frequently as larvae in the previous investigations in the province (35–37). *Aedes vexans* was the most abundant species (Table 1) mostly captured in the second year of study (2014) adjoining Anzali Wetland. This species is important as a vector of WNV, Snowshoe Hare virus (SSHV) and Tahyna virus (TAHV) (Bunyaviridae: *Orthobunyavirus*) (2, 4). The second prevalent species which collected in the current study was *Cx. tritaniorhynchus* (Table 1) is an important vector of WNV and JEV (4, 21). *Culex pipiens* was found in all collected sites (Table 1). This species is a domestic mosquito which has a role in the transmission of some arboviruses including WNV, TAHV and SINV (2, 4). *Culex theileri* was collected only in 2014 from Anzali Wetland (Table 1). The species is known the vector of WNV (4). Some insect-specific flaviviruses (ISFs) were isolated from this species in Portugal (38) and Turkey (39) and designated *Culex theileri* flavivirus (CTFV). *Coquilletidia richiardii* was also found (Table 1), which is considered among the vectors of WNV, SINV and Batai Virus (BATV) (Bunyaviridae: *Orthobunyavirus*) (2, 4). Among the aforementioned species, *Cq. richiardii* and *Cx. Pipiens* in Europe and *Cx. tritaeniorhynchus* in Asia are the main vectors of WNV (4). Among anopheline mosquitoes there are some reports of isolation of WNV and BATV from *An. maculipennis* s.l. (2, 4), which also was collected during this study.

Favorable climate of the region, including high precipitations, provides conditions that can lead to the emergence or re-emergence of mosquito-borne diseases (Fig. 2). The presence of rice fields, wetlands and lagoons used by migratory birds may cause new viral outbreaks (31). The area also provides suitable larval habitats for many mosquito species. Guilan Province is a well-known touristic place with thousands of visitors annually from different parts of the country. That can increase probable imported cases of mosquito-borne infections. Also the important vectors of WNV, *Cx. pipiens* and *Cx. tritaeniorhynchus*, are among the most abundant and widespread species in the province (37).

As far as the authors know this study is the first one with the aim of *Flavivirus* screening in mosquitoes in Guilan Province. Most of the available data about the mosquitoes of the province are based on larval collections (35–37). Sampling adult mosquitoes in Guilan Province, using light traps and hand catch, can be another advantage of the present investigation. The collection, anaesthetizing, identification, and preparing of collected specimens based on molecular protocols are time-consuming. That effected sample size which could be one of the limitations of the present study to detect the virus. Using sentinel birds as bait may increase the possibility of sampling ornithophilic species which are WNV vectors and detect the virus. That was not among the goals of this study, however that may be used in forthcoming surveys. In view of the absence of official vector surveillance for WNV and many other mosquito-borne viruses in Iran, such investigations should be continued.

## Conclusion

Although, the mosquito species found in this survey are among proven or potential *Flavivirus* vectors worldwide, our screening by RT-PCR did not reveal any *Flavivirus* products. The total sample size could effect the outcomes. Despite this fact surveillance should be continued due to the presence of WNV infections in humans, horses, birds and known vectors in the region. The probable forthcoming finding of WNV in mosquitoes will make a chance to compare the relativeness of detected virus strains in vectors and vertebrate hosts in the region. Also screening for other arboviruses other than Flaviviridae, which were not investigated in the present study, should be considered for future studies.
